# The impact of a multicomponent-functional training with postural correction on functional balance in the elderly with a history of falling

**DOI:** 10.1186/s40634-022-00459-x

**Published:** 2022-03-01

**Authors:** Parisa Sedaghati, Maryam Goudarzian, Somayeh Ahmadabadi, Seyed Mojtaba Tabatabai-Asl

**Affiliations:** 1 Department of Sports Injuries and Corrective Exercise, Faculty of Physical Education and Sport Sciences, University of Guilan, Rasht, IR Iran; 2 Iranian Research Center On Healthy Aging, Sabzevar University of Medical Sciences, Sabzevar, IR Iran; 3 Department of Physical Education and Sports Sciences, Farhangian University, Tehran, IR Iran

**Keywords:** Aging, Fall, Intervention, Physical activity

## Abstract

**Purpose:**

Good posture plays a significant role for the elderly in achieving optimal quality of life. This study aimed to evaluate the impact of multicomponent functional training with postural correction on functional balance in the elderly with a history of falling.

**Methods:**

This study was a randomized controlled, single-blind study. Subjects (*n* = 28, mean age = 70 years) with a history of falling were selected and randomly allocated to either a multicomponent functional training (*n* = 14) or a control group (*n* = 14). The experimental group exercised for 8 weeks, three days per week for 60 min per day. The training program with strength, endurance, and balance parts was conducted in the multi-task conditions to stimulate the physical and cognitive abilities focusing on the attentional-correct posture. The control group received conventional care. The Berg balance and short physical performance battery tests were used in the pre-test and post-test. The adjusted post-test means of experimental and control groups were analyzed using the ANCOVA test to eliminate any pretest effects.

**Results:**

This study found a significant effect of training on Berg balance test (*P* = 0.001), Timed Up and Go with D-T (*P* = 0.01), Timed Up and Go (*P* = 0.002), and Short Physical Performance Battery (*P* = 0.001).

**Conclusions:**

Eight weeks of multicomponent exercise training has beneficial effects on balance and physical function and results in improved equilibrium and a decreasing probability of falling. Therefore, practitioners can use this 8-week training program for older adults.

## Introduction

One-third of the elderly with age over 65 years living in urban experience falls each year in China [12]. Falls account for over 80% of hospitalizations for patients aged 65 years or over [26]. Several risk factors for falling include balance impairment, decreased muscle strength, and gait impairment [44]. Interventions that address multiple risk factors have demonstrated a significant reduction in falls among community-dwelling older adults [21].

Reduced mobility is considered one of the main predictors of falling in older adults [8]. Hence, it seems very important to introduce a fall prevention program that targets mobility for such age groups, especially for those living in nursing and residential care facilities [30]. Otherwise, the percentage of older people staying in nursing homes would also significantly increase. In care homes, older adults constitute a diverse and heterogeneous community with a high prevalence of dependence in everyday life tasks, cognitive disability, depression, a high fall incidence, multi-morbidity, and poly-medication [15]. Besides, old people living in nursing homes for a long time often tend to be highly inactive, participating in sedentary activities for most of the day [5].

The cause of falls is multifactorial, with one major factor being compromised postural function. Postural control is the ability to control the body position in space during standing and walking tasks for stabilization and orientation purposes [39].

Dual-task efficiency refers to the ability to execute two tasks concurrently with the postural double-task management relating to situations when the postural control requires at least one of the tasks, such as walking while talking on the phone, or carrying a bowl or cup [40]. Deficiency in dual-task postural control is associated with decreased cognitive performance in older adults [25] and an elevated rate of falls in the elderly [32]. Some everyday life activities are multitasking activities that generate conflicting demands on attention and involve challenging tasks and cognitive functions [34]. While attention is limited and demands are higher than capacity, a dual-task performance might be affected in a single-task performance compared to the performance of the same tasks [2]. For accomplishing daily activities independently, altered management of attention capital is considered [42]. For evaluating this relationship, much research has focused on the interaction between postural control and using the dual-task postural control paradigms [6].

In recent years, many studies have shown that old age is no deterrent to improving coordination, power, and autonomous transfer by introducing adapted physical activity [13]. In a systematic and meta-analysis, findings showed that various balance training modalities lead to changes in static/dynamic steady-state, constructive, and reactive balance assessments as well as in the efficiency of balance test batteries in stable older people [28]. A successful balance training program for healthy older adults consists of 11–12 weeks, three sessions per week for a total of 36–40 sessions, 31–45 min per session, and 91–120 min of balance training per week [28]. While Agmon et al. indicated prospective approaches to strengthen postural control, dual-task management should provide centered dual-task preparation and resolve activities that are more associated with fall risk [1]. Therefore, Agmon et al. stated for evaluating the best appropriate protocol, prospective studies may also concentrate on motor learning features that can extend the retention of dual-task training benefits [1]. The novelty of this study refers to the multi-component functional training (MCFT) with components of functional balance and lower-extremity muscle strength affecting postural correction during physical activity and also functional balance assessment in single-task and dual-task conditions.

Therefore, this study aimed to evaluate the impact of multicomponent-functional training with postural correction on functional balance and the probability of falling in the elderly living in nursing homes with a history of falling.

## Materials and methods

### Participants

**Fig. 1:**
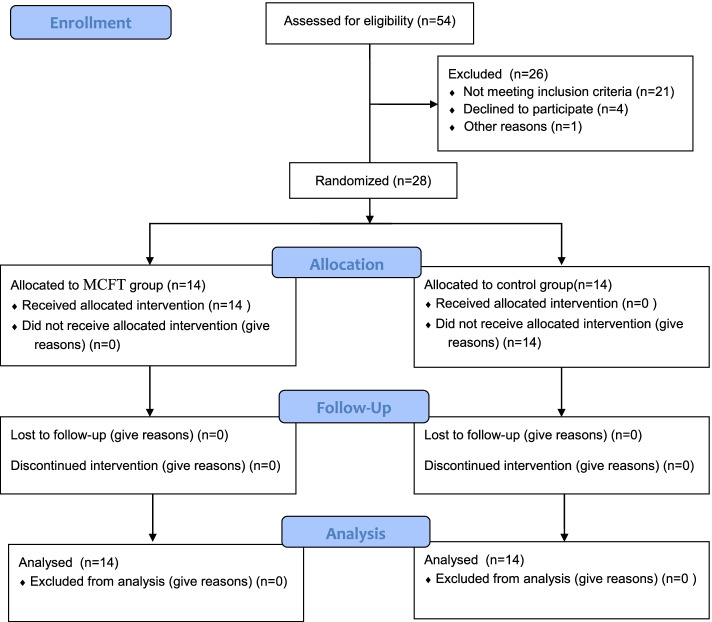
Diagram of randomized control trial

Participants included 28 males recruited from Elderly Care Center (age = 70.8 ± 2.5 years, Weight = 66.6 ± 2.3 kg, height = 167.9 ± 2.4 cm, BMI = 23.6 ± 1.1). Participants were randomly allocated to either multicomponent functional training (MCFT) or control group (CG). All the participants completed and signed informed consent, and all ethical considerations were observed based on the Helsinki Declaration. Figure 1 shows the CONSORT flowchart of the study and the allocation procedure of subjects to groups. Also, the reporting checklist for randomized trials based on CONSORT guidelines can be found in Appendix 1.

### Procedure

In this randomized-controlled study, the inclusion criteria were as follows: 1) age ≥ 68 years old, 2) Farsi Version of the Mini-Mental State Examination (MMSE) > 24 [20], 3) walking or accomplishing other daily activities without assistance, 4) being healthy without any acute or chronic diseases or physical, mental, psychological, and other disorders (e.g., cardiovascular, respiratory, skin problems, osteoarthritis), 5) the ability to participate in regular training sessions (not missing more than two sessions during the entire research period), 6) no history of regular physical activity in the 6 months before participating in the study, 7) the ability to stand for at least one minute and walk 10 m unaided, 8) normal vision (or corrected vision).

Exclusion criteria were as follows: 1) a history of depression or other psychological disorders, 2) orthopedic problems or severe lower/upper extremity deformities, 3) a history of lower limb joint replacement, 4) a history of balance disorders and recurrent positional vertigo, 5) severe pain in lower/upper extremities, 6) vestibular diseases, 7) a severe visual impairment, 8) unwillingness to continue participation in the study, 9) missing three consecutive training sessions. Participants were not eligible whenever the medical staff realized they were clinically unstable or showed no inclination to continue the study [1, 35, 38]. All participants were informed of the purpose and possible risks involved in the research study and were required to read and sign an informed consent form before participation. sand the study was registered in the Iranian Registry of Clinical Trials (IRCT20160815029373N5).

### Randomization

After the primary assessment, the participants will be randomly (lottery method) allocated into either MCFT or CG group in a double-blinded design for both the participants and assessors.

### Exercise intervention

#### MCFT

Practicability of the MCFT were assessed by a pilot study with full details of volume, intensity, and the type of strength and balance exercises using the dual-task training [1, 4, 33, 35, 36]. Participants allocated to this MCFT group were attended a training program, three times a week for an hour per session. The program consisted of strength and balance exercises performed and thought by an experienced exercise physiologist. This point should be mentioned that participants must maintain correct posture during all types of physical activity. Also, participants continue their daily activities as usual.

In the multicomponent functional training (MCFT), physical activities were designed based on executive functions and conducted alongside approximately four MCFT. The difficulty of dual-tasks increased by augmenting the complexity of motor tasks mentioned in Table1.

**Table 1:** MCFT Program for the 8 weeks and progression of the complexity of secondary tasks [4, 33]

Phases program	First month	Second month
Type of physical activity	Strength Static balance + dual-task + corrective posture^a^	Strength + Dynamic balance + dual-task + corrective posture^a^
1-Warm-up 5 min	Range of motion for different joints	
2-Strength lower extremity: Chair squat and stand, Leg flexion, Leg extension, Leg abduction, Hip extension, Standing on the tips of toes and heels	4–5 ex: 2 sets, 8 rep	4–5 ex:1–2 sets, 12 rep
3-Balance: Feet-together stance, legged stand, Semi-tandem/Tandem balance, Circuit training, Gait training-stairs & obstacle function	2–3 ex, progressive difficulty in sitting position and progressing to standing position	4–5 ex, progressive difficulty in standing position with decreasing arm support and increasing instability with foam mat
4- Dual-task (cognitive function): One type of cognitive exercise in each session) Naming colors/days of the week/names, Counting by twos starting 0 till a number ≤ 30, Counting backward by ones(	In 2–3 of strength ex and 1–2 of balance ex	In 1–2 of strength ex and 3–4 of balance ex
5-Cool down 5 min	Stretching, breathing exercises	

ex exercises, rep repetitions

^a^Participants must maintain correct posture during all types of physical activity

Anthropometric measurements

Outcome Variable

The primary outcome measures presented in Table 2 consist of Short Physical Performance Battery (SPPB), Berg balance test (BBT), Timed Up and Go (with and without D-T), tandem gait test, and anthropometric measurements: weight, height, body mass index [18]. Height and weight were measured to the nearest 0.1 cm and 0.1 kg, respectively.

**Table 2:** Tests to assess the functional abilities

Test	Parameters	Description
Berg balance test	Postural stability	Performance of 14 functional tasks [7]. The inter-rater reliability was also high, with a pooled estimate of 0.97[17]
Timed Up and Go (with and without D-T)	Functional balance	Get up from a chair, walk 3 m at a normal pace, turn around, walk back to the chair and sit down again [31, 35], participant test was carried out by carrying a ball [1, 14]. The test–retest reliability (ICC) ranged from 0.54 to 0.85[11]
Short Physical Performance Battery	Lower extremity function: static balance, gait speed, and getting in and out of a chair	Side-by-side, semi-tandem, and tandem stands (10 s); 4 m walk test at a comfortable speed and 5 quickly sit to stand from a chair without upper extremity assistance [1, 24]. test–retest reliability of the SPPB was high: 0.87 for subjects aged 65 to 74 years[23]
tandem gait test(TGT)	Dynamic gait balance	to take 12 consecutive steps with the feet aligned heel to toe in tandem on a straight line with their eyes open (stopwatch), without walking aids, and with their arms hanging by the sides of their body [27]. Reliability coefficients were 0.62 for tandem gait for the disabled sample[22]

### Power and sample size

The sample size was calculated using G * Power software which evaluated an exercise program with TUG performance of 28 subjects, with a mean time to perform TUG before and after the intervention of 17.92 ± 9.42 and 16.35 ± 7.55 s, respectively. Given a *P*-value of 0.05, a power of 80% [3], the TUG test time in seconds, and an effect size of 0.7 for the minimal clinically significant differences, 14 volunteers would be needed in each group (Fig. 1).

### Statistical analysis

Statistical analysis was performed using the software SPSS version 16. For assessing normality of distribution and comparison of demographic variables, the Shapiro–Wilk test, and the independent sample t-test were performed respectively. For excluding any possible pre-test effect, the adjusted post-test means were tested using ANCOVA and for comparison of the means of within-group between the pre-/post-test, the paired t-test was used. The level of significance was fixed at 0.05 and 0.01 level.

## Results

The general demographic characteristics of the 28 subjects are showen in Table 3 and there were no significant differences between groups in examined variables (*P* > 0.01). The independent t-test revealed no significant difference at pre-test in the demographic data between the two groups(*P* > 0.01).

**Table 3:** Comparing the demographic data of the participants between the experimental and control groups

Group	EG (*n* = 14, F = 7;M = 7) Mean ± SD (range)	CG (*n* = 14, F = 7;M = 7) Mean ± SD (range)	*P*
Age (y)	70.42 ± 2.70	71.07 ± 2.26	NS
Height (cm)	168.42 ± 2.40	167.35 ± 2.23	NS
Weight (kg)	67.12 ± 5.19	66.05 ± 2.37	NS
BMI	23.68 ± 1.21	23.59 ± 1.11	NS
MMSE (score)	26.08 ± 1.25	26.33 ± 1.37	NS
Fall number	2.62 ± 0.50	2.69 ± 0.48	NS

*EG* Experimental Group, *CG* Control Group, *NS* non-significant, *MMSE* Mini-Mental State Examination

As shown in Table 4. the paired t-test of BBS, TUG, TUG-D, TG, and SPPB in the control group revealed no significant difference between the pre-/post-test (*P* ≥ 0.01). However, the paired t-test showed a significant difference between the pre-/post-test (*P* = 0.001) for the effects of MCFT on BBS, TUG, TUG-D, TG, and SPPB.

**Table 4:** The Pre and Post Test Means of Experimental and Control Groups (*n* = 28)

Groups	E G (*n* = 14)				C G(*n* = 14)			
	**PreT**	**PostT**	**T**	***P***	**PreT**	**PostT**	**T**	***P***
**BBS**	27.00 ± 2.18	31.00 ± 3.13	-7.95	0.001^a^	26.00 ± 1.79	25.64 ± 1.90	0.92	0.373
**TUG(s)**	15.11 ± 1.36	13.87 ± 1.64	5.54	0.001^a^	14.86 ± 1.60	15.05 ± 1.60	-1.24	0.236
**TUG-Dual(s)**	16.33 ± 1.47	15.39 ± 1.67	5.94	0.001^a^	16.32 ± 1.60	16.37 ± 1.62	-0.61	0.548
**TG**	6.35 ± 1.00	8.00 ± 1.24	-8.25	0.001^a^	6.14 ± 1.23	6.50 ± 1.09	-0.36	0.720
**SPPB**	6.42 ± 0.85	8.21 ± 1.25	-8.33	0.001^a^	6.28 ± 1.20	6.35 ± 1.15	-1.58	0.136

*EG* Experimental Group, *CG* Control Group, *PreT* Pre Test, *PostT* Post Test, *BBS* Berg Balance Scale, *TUG* Timed Up and Go test, *TG* tandem gait, *SPPB* Short Physical Performance Battery test, ^a^Statistically significant

As shown in Table 5, the ANCOVA test with covariate pre-test revealed a significant difference in BBS (*P* = 0.001), TUG (*P* = 0.018), TUG-D (*P* = 0.01), TG (*P* = 0.002), and SPPB (*P* = 0.001) between post-test of experimental and control groups.

**Table 5:** Analysis of Covariance for the Selected Variables among Experimental Group & Control Groups (*n* = 28)

Variables	Type III Sum of Squares	F	Sig	Partial Eta Squared
BBS	87.87	35.08	0.001^a^	0.626
TUG(s)	3.38	6.62	0.018	0.240
TUG-Dual(s)	1.69	8.09	0.01^a^	0.278
TG	8.11	12.69	0.002^a^	0.377
SPPB	7.62	14.72	0.001^a^	0.412

*BBS* Berg Balance Scale, *TUG* Timed Up and Go test, *TG* tandem gait, *SPPB* Short Physical Performance Battery test, ^a^Statistically significant.

## Discussion

The current study explored the effect of a MCFT program on balancing single- and dual-tasks, including gait and cognitive tasks, in elderly subjects with a history of falling while residing in nursing homes.

This study found a significant effect of MCFT on BBS, TUG, TUG-D, TG, and SPPB between the pre-/post-test and the exercise and control groups.

Considering the exponentially rising number of people over 68, there is a lack of recommendations for the geriatric population and practitioners working in this sector. Older adults are at specific risk of adverse effects in long-term nursing homes where they have been the subject of strategies to avoid or reverse frailty [46].

Dual-task training was introduced as functional training focusing on physical parameters and cognitive variables in the elderly [19, 29]. Dual-task paradigms were widely used to evaluate the degree of automatic and controlled handling of postural stability among different age groups [10]. The present study used a previously published protocol on dual-tasks which was applicable and it showed improvements in many functional outcomes [19, 29, 35, 37]. Besides, the proposed interventions are easy to apply and include extensive practical issues on application (e,g., training frequency, volume, intensity, individualization, and rest intervals). These training regimes are easy to use in long-term nursing homes. Thus, current findings provide valuable insight into the impact of the dual-task program on patients living in long-term in a nursing home that integrates physical and cognitive factors consistent with aging. Moreover, analysis of an MCFT program and the same program with concurrent cognitive training, or dual-task, supported us to design the interventions to improve or at least maintain functionality and cognition in long-term nursing home peoples. In this regard, Rezola-Pardo et al. reported the effects of the dual-task program on people living in long-term in a nursing home, taking overall physical, cognitive, and emotional variables linked to frailty [35]. Furthermore, the analysis of an MCFT program and the same program with concurrent postural training, dual-task, supported our findings to design the interventions to improve or maybe maintain functionality in nursing home peoples [35].

Comparison our findings with recently published articles on young healthy adults, it seems that the effect of dual-task protocols is independent of age [28]. Given the limited number of training protocols [28], further investigation is necessary to prove and specify preliminary dosage–responding relationships of dual-task physical training protocols in healthy older adults.

Critically, the review by Zijlsra et al. (2008) questioned the added value of dual over single postural task conditions for fall prediction [47]. Stins and Beeks (2012) also expressed reservations about the possibility for the cognitive processes to influence postural control [41]. Moreover, Agmon et al. reported no improvement in transfer between single-task and dual-task performance [1].

Most studies evaluating postural performance in the static standing revealed a difference in performance between healthy older adults and young adults [45] that may be related to a greater incidence of falls in older adults as an indicator of declined postural stability [9].

On the other side, considering a relation between dual-task training and fall, Agmon et al. suggested that future studies should focus on dual-task training and report tasks that have the highest correlation with the risk of falling to improve dual-task postural control. Moreover, a long-term follow-up of fall occurrences and daily activity should apply to provide a better understanding of whether improved dual-task postural control impacts these factors [1]. Furthermore, future research should focus on motor learning elements that may extend the retention of dual-task training benefits to determine the most effective protocols. We believe that several inconsistencies could be resolved by a careful selection of dual-task studies based on the methodological criteria. In line with the improvement in the probability of falling, Tabatabai et al. (2021) studied the impact of combined Cawthorne-Cooksey exercises on functional balance and fall probability in elderly people and their results showed a significant improvement in balance and reduced risk of falling in the adult people [43]. Also, the results of the present study are in line with the study of Długosz et al (2021). They investigated the effects of three months of pilates training on balance and fall risks in older women. Their results showed statistically significant improvements in balance and risks of falling in old people [16].

To the best of our knowledge, no study has explored the impacts of a managed dual-task MCFT in the elderly with a history of falling and living nursing home facilities and assessing functional capacity under both single- and dual-task conditions and physical activity. Moreover, we mention possible limitations of the present study. The selected inclusion criteria excluded the majority of long-term nursing home residents, as we included light to moderately dependent participants, while the prevalent profile in this type of institution is strictly dependent. Therefore, we might come across difficulties in reaching the anticipated sample size. However, the large number of agreements made with long-term care centers with the general office. Welfare Organizations will facilitate the recruitment of sufficient subjects.

## Conclusions

In summary, the present study indicated that a selective posture-corrected multicomponent exercise with a single or dual-task can improve the functional balance in elderly residents in a nursing home with a history of falling.

## Data Availability

All raw data sets associated with the paper will be available on request.

## Appendix

Table

**Table 6:** CONSORT 2010 checklist of information to include when reporting a randomize trial

Section/Topic	Item No	Checklist item	Reported on page No
**Title and abstract**			
	1a	Identification as a randomised trial in the title	N/Y
	1b	Structured summary of trial design, methods, results, and conclusions (for specific guidance see CONSORT for abstracts)	1
**Introduction**			
Background and objectives	2a	Scientific background and explanation of rationale	1–3
	2b	Specific objectives or hypotheses	3
**Methods**			
Trial design	3a	Description of trial design (such as parallel, factorial) including allocation ratio	4
	3b	Important changes to methods after trial commencement (such as eligibility criteria), with reasons	4
Participants	4a	Eligibility criteria for participants	4
	4b	Settings and locations where the data were collected	3–4
Interventions	5	The interventions for each group with sufficient details to allow replication, including how and when they were actually administered	5–6
Outcomes	6a	Completely defined pre-specified primary and secondary outcome measures, including how and when they were assessed	6
	6b	Any changes to trial outcomes after the trial commenced, with reasons	N/Y
Sample size	7a	How sample size was determined	5
	7b	When applicable, explanation of any interim analyses and stopping guidelines	N/Y
Randomisation:			
Sequence generation	8a	Method used to generate the random allocation sequence	5
	8b	Type of randomisation; details of any restriction (such as blocking and block size)	N/Y
Allocation concealment mechanism	9	Mechanism used to implement the random allocation sequence (such as sequentially numbered containers), describing any steps taken to conceal the sequence until interventions were assigned	5
Implementation	10	Who generated the random allocation sequence, who enrolled participants, and who assigned participants to interventions	5
Blinding	11a	If done, who was blinded after assignment to interventions (for example, participants, care providers, those assessing outcomes) and how	N/Y
	11b	If relevant, description of the similarity of interventions	N/Y
Statistical methods	12a	Statistical methods used to compare groups for primary and secondary outcomes	7
	12b	Methods for additional analyses, such as subgroup analyses and adjusted analyses	7
**Results**			
Participant flow (a diagram is strongly recommended)	13a	For each group, the numbers of participants who were randomly assigned, received intended treatment, and were analysed for the primary outcome	7
	13b	For each group, losses and exclusions after randomisation, together with reasons	7
Recruitment	14a	Dates defining the periods of recruitment and follow-up	7–8
	14b	Why the trial ended or was stopped	N/Y
Baseline data	15	A table showing baseline demographic and clinical characteristics for each group	7–8
Numbers analysed	16	For each group, number of participants (denominator) included in each analysis and whether the analysis was by original assigned groups	7–8
Outcomes and estimation	17a	For each primary and secondary outcome, results for each group, and the estimated effect size and its precision (such as 95% confidence interval)	7–8
	17b	For binary outcomes, presentation of both absolute and relative effect sizes is recommended	N/Y
Ancillary analyses	18	Results of any other analyses performed, including subgroup analyses and adjusted analyses, distinguishing pre-specified from exploratory	N/Y
Harms	19	All important harms or unintended effects in each group (for specific guidance see CONSORT for harms)	N/Y
**Discussion**			
Limitations	20	Trial limitations, addressing sources of potential bias, imprecision, and, if relevant, multiplicity of analyses	10
Generalisability	21	Generalisability (external validity, applicability) of the trial findings	8
Interpretation	22	Interpretation consistent with results, balancing benefits and harms, and considering other relevant evidence	8–10
**Other information**			9–10
Registration	23	Registration number and name of trial registry	12
Protocol	24	Where the full trial protocol can be accessed, if available	6
Funding	25	Sources of funding and other support (such as supply of drugs), role of funders	12

^6^

## Abbreviations

Ex: Exercises
Rep: Repetitions
NS: Non-significant
MMSE: Mini-Mental State Examination
EG: Experimental Group
CG: Control Group
PreT: Pre Test
PostT: Post Test
BBS: Berg Balance Scale
TUG: Timed Up and Go test
TG: Tandem gait
SPPB: Short Physical Performance Battery test
